# Supplementary material for: Electronic control of redox reactions inside Escherichia coli using a genetic module
Source: PLoS One. 2021 Nov 18;16(11):e0258380. doi: 10.1371/journal.pone.0258380 (PMC8601525; doi:10.1371/journal.pone.0258380)
Supplement: S1 Methods — (DOCX) [file pone.0258380.s001.docx]

# **S1 Methods**

**Analysis of redox processes using cyclic voltammetry**

The bioreactor was prepared as previously described up until the step in which the potential was switched to -0.56 V_Ag/AgCl_. At this time, cyclic voltammetry was conducted before fumarate, nitrate or nitrite addition. Cyclic voltammograms were recorded in 5 cycles from −0.8 V_Ag/AgCl_ to +0.8 V_Ag/AgCl_ at a scan rate of 1 mV/s. After the measurement, the electrode was switched to chronoamperometry.

**Analysis of cytochromes c using enhanced chemiluminescence**

To analyze the abundance of c-type cytochromes in different *E. coli* strains, whole cell lysates were first prepared under conditions which preserve the bond between a heme *c* and cysteine residue in the protein backbone. Cells from 1.5 mL of culture were harvested by centrifugation and immediately frozen at -20°C for later analysis. Immediately before analysis, the cell pellets were thawed and resuspended in 0.1 mL Bacterial Protein Extraction Reagent (B-Per, ThermoScientific, Grand Island, NY). The cells were then lysed by addition of 6 μg mL^-1^ chicken egg white lysozyme (Sigma), 1 μg mL^-1^ DNAase, 3.9 mM MgSO_4_, 0.96 mM EDTA, and 0.98 mM phenylmethylsulfonyl fluoride and incubation for 30 minutes at room temperature. The total protein concentration of the resulting whole cell lysates was determined by BCA Protein Assay Kit (ThermoScientific), and the whole cell lysates were diluted in 100 mM HEPES, pH 7.4, to yield lysates with equal total protein concentrations. Whole cell lysates were then separated by SDS-PAGE under non-reducing conditions, transferred to nitrocellulose, and the peroxidase activity of hemes *c* was detected by chemiluminescence. Whole cell lysates were diluted into NuPAGE 4x Sample Buffer (Bio-Rad) and heated at 95 °C for 5 minutes. For each strain, a total of 8 µg protein was loaded into a lane of a 4-20% Tris-HCl polyacrylamide gel (Bio-Rad) and separated by electrophoresis at 200 V for 1 hour. The gel was rinsed twice in water and then equilibrated in a cold Pierce Western Transfer buffer (ThermoScientific) for 15 minutes. The proteins were transferred to a 0.45 μm nitrocellulose membrane (Bio-Rad, Hercules, CA) at 2.5 A 25 V for 7 minutes with Trans-Blot Turbo Transfer System (Bio-Rad). Ponceau S staining was used to confirm uniform transfer across all lanes. The nitrocellulose membrane was incubated for 5 minutes in 10 mL of Pierce Pico West Enhanced Chemiluminescence substrate (ThermoScientific), a 1:1 mixture of Pico West Peroxide Solution and Luminol Enhancer solution. The chemiluminescence signal, arising from protein-bound metals, was detected using the FluorChem E system.

**Determination of colony-forming units (CFU)**

The colony forming units (cfu) concentration for each culture was measured by preparing a serial dilution of the samples in sterile PBS. 100 µL of each relevant dilution was spread on LB-agar plates with antibiotics. The plates were grown aerobically at 37 °C and the colonies were counted the next day.

**Supporting References**

[Boyarskiy, S., Davis López, S., Kong, N., & Tullman-Ercek, D. (2016). Transcriptional feedback regulation of efflux protein expression for increased tolerance to and production of n-butanol. *Metabolic Engineering*, *33*, 130–137.](http://paperpile.com/b/DTFsK9/WoD8q)

[Cherepanov, P. P., & Wackernagel, W. (1995). Gene disruption in *Escherichia coli*: TcR and KmR cassettes with the option of Flp-catalyzed excision of the antibiotic-resistance determinant. *Gene*, *158*(1), 9–14.](http://paperpile.com/b/DTFsK9/Sx1WJ)

[Goldbeck, C. P., Jensen, H. M., TerAvest, M. A., Beedle, N., Appling, Y., Hepler, M., Cambray, G., Mutalik, V., Angenent, L. T., & Ajo-Franklin, C. M. (2013). Tuning promoter strengths for improved synthesis and function of electron conduits in *Escherichia coli*. *ACS Synthetic Biology*, *2*(3), 150–159.](http://paperpile.com/b/DTFsK9/iOkSs)

[Jensen, H. M., Albers, A. E., Malley, K. R., Londer, Y. Y., Cohen, B. E., Helms, B. A., Weigele, P., Groves, J. T., & Ajo-Franklin, C. M. (2010). Engineering of a synthetic electron conduit in living cells. *Proceedings of the National Academy of Sciences of the United States of America*, *107*(45), 19213–19218.](http://paperpile.com/b/DTFsK9/6uVd2)

[Jensen, H. M., TerAvest, M. A., Kokish, M. G., & Ajo-Franklin, C. M. (2016). CymA and Exogenous Flavins Improve Extracellular Electron Transfer and Couple It to Cell Growth in Mtr-Expressing *Escherichia coli*. *ACS Synthetic Biology*, *5*(7), 679–688.](http://paperpile.com/b/DTFsK9/9qMUc)

Dumon-Seignovert L., Cariot G., and Vuillard L. (2004). Protein Expression and Purification 37, 203-206
