# Supplementary material for: Electronic control of redox reactions inside Escherichia coli using a genetic module
Source: PLoS One. 2021 Nov 18;16(11):e0258380. doi: 10.1371/journal.pone.0258380 (PMC8601525; doi:10.1371/journal.pone.0258380)
Supplement: S1 Table — (PDF) [file pone.0258380.s002.pdf]

Table S1. Strains used in this study.

Nickname	Description	Strain #	Genotype	Source
E.coli	*E. coli* parent strain	-	C43(DE3)	Dumon-Segnoivert et al, 2004
Ccm-*E.coli*	*E. coli* expressing *ccmABCDEFGH* and carrying an empty Kan vector	MFe408	C43(DE3) +pEC86, pSBIET2	Goldbeck et al. 2013.
Mtr-*E.coli*	*E. coli* expressing IPTG-inducible *mtrCAB*	MFe409	C43(DE3) +pEC86, I5023	Goldbeck et al. 2013.
CymAMtr-*E.coli*	*E. coli* expressing IPTG-inducible *cymAmtrCAB*	MFe444	C43(DE3) +pEC86, I5049	Jensen et al. 2016.
CymAMtr- *Δfrd*	*E. coli* expressing IPTG-inducible *cymAmtrCAB* in the *ΔfrdABCD* background	MFe1085	C43(DE3) *ΔfrdABCD* +pEC86, I5049	This study
CymAMtr-*ΔfrdΔsdh*	*E. coli* expressing IPTG-inducible *cymAmtrCAB* in the *ΔfrdABCD ΔsdhABCD* background	MFe1086	C43(DE3) *ΔfrdABCD ΔsdhABCD* +pEC86, I5049	This study
CymAMtr^{S}-*ΔfrdΔsdh*	*E. coli* expressing *cymAmtrCAB* under control of the *epcD* promoter in the *ΔfrdABCD ΔsdhABCD* background	MFe854	C43(DE3) *ΔfrdABCD ΔsdhABCD* +pEC86, I5105	This study
CymAMtr^{S}-*frd*$^{+}$*Δsdh*	*E. coli* expressing *cymAmtrCAB* under control of the *epcD* promoter and *frdABCD* in the *ΔfrdABCD ΔsdhABCD* background	MFe1089	C43(DE3) *ΔfrdABCD ΔsdhABCD* + I5105+pEC86+pAF -frdABCD	This study
CymAMtr-*ΔmenA*	*E. coli* expressing IPTG-inducible *cymAmtrCAB* in the *ΔmenA* background	MFe1083	C43(DE3) *ΔmenA* +pEC86, I5049	This study
CymAMtr-*ΔmenC*	*E. coli* expressing IPTG-inducible *cymAmtrCAB* in the *ΔmenC* background	MFe1084	C43(DE3) *ΔmenC* +pEC86, I5049	This study
CymAMtr-*menC*$^{+}$	*E. coli* expressing IPTG-inducible *cymAmtrCAB* and *menC* in the *ΔmenC* background	MFe1128	C43(DE3) *ΔmenC* +pEC86, I5049, pAF-menC	This study

Ccm-*ΔnuoH*	*E. coli* constitutively expressing *ccmABCDEFGH* in the *ΔnuoH* background	MFe1088	C43(DE3) *ΔnuoH* +pEC86, pSB1ET2	This study
CymAMtr-*ΔnuoH*	*E. coli* expressing IPTG-inducible *cymAmtrCAB* in the *ΔnuoH* background	MFe1087	C43(DE3) *ΔnuoH* +pEC86+I5049	This study
