# Supplementary material for: Electronic control of redox reactions inside Escherichia coli using a genetic module
Source: PLoS One. 2021 Nov 18;16(11):e0258380. doi: 10.1371/journal.pone.0258380 (PMC8601525; doi:10.1371/journal.pone.0258380)
Supplement: S2 Table — (PDF) [file pone.0258380.s003.pdf]

Table S2. Plasmids used in this study.

Plasmid	Description	Source
pEC86	Constitutive expression of *ccmABCDEFGH*	Goldbeck et al. 2013 (Goldbeck et al., 2013)
I5049	IPTG-inducible expression of *cymAmtrCAB*	Jensen et al 2016 (Jensen et al., 2016)
I5023	IPTG-inducible expression of *mtrCAB*	Jensen et al 2010 (Jensen et al., 2010)
I5105	expression of *cymAmtrCAB* under control of Stress-responsive promoter *epcD* promoter	This study
pSB1ET2	*Empty backbone of I5049*	Jensen et al 2010 (Jensen et al., 2010)
pAF-frdABCD	*Expression of frdABCD*	This study
pCP20	*Expression* the *Flp* recombinase	Cherepanov et al 1995 (Cherepanov & Wackernagel, 1995)
pAF-MenC	*Expression of menC*	This study
