# Supplementary material for: Electronic control of redox reactions inside Escherichia coli using a genetic module
Source: PLoS One. 2021 Nov 18;16(11):e0258380. doi: 10.1371/journal.pone.0258380 (PMC8601525; doi:10.1371/journal.pone.0258380)
Supplement: S3 Table — (PDF) [file pone.0258380.s004.pdf]

Table S3. Primers Used in This Study, Related to the Experimental Procedures.

Primer name	Sequence 5’ -> 3’	Reference
sdh-pKD3 fw	'cgatagcgtccattctccatcgcgtttccggtgtgatcacctttgttgcaggtgtaggctggagctgcttc'	This study
sdh-pKD3 rev	'gatggcgcgcgtcgggttcagccccttcggacatacactgacgcagttcaatgggaattagccatggtcc'	This study
sdh-rev	‘atgcttacttcgccgtggat’	This study
sdh-fw	‘tgaacagcctatactgccgc’	This study
nuoH-pKd3 fw	'ctgaccatcctcaaagcggtggtgatcctgctggtggttgtcacctgcgggtgtaggctggagctgcttc'	This study
nuoH-pKd3 rev	'tattgcgcctgccagagaatgacagccgccgttaccagcaagttgatcagatgggaattagccatggtcc'	This study
nuoH- fw	tccggtgctggctggcgcgcatcttgagga	This study
nuoH- rev	gcaggccgatcatccagata	This study
frdABCD+RBS – fw (GB)	'aataacaagttgataacaagctagccaaaaacaacattctaacta'	This study
frdABCD – rev (GB)	'ttcgttttatttgatgcctggttattttacattggcgatgcgtta'	This study
pMCC(SmaI)-crispr.fwd	‘atacgcaaaccgcctctccccatggatcctatttcttaataactaaaaatatgg'	This study
pMCC(SmaI)-crispr.rev	'gaagaacccttccagtgccccactgagacttgttgagttgaattcatg'	This study
Crisper frdA-for	'gtgcaaacctttcaagccga'	This study
Crisper frdD-rev	'aggatagcagccagaccgta'	This study
menA-pKd4-fw	‘atgactgaacaacaaattagccgaactcaggcgtggctggaaagtttacgaccgtgt’	This study
menA-pKd4-rev	‘ttatgctgcccactggcttaggaatatccctaaaacaaacagcaggttagtatggga’	This study
menA-fw	‘tatttgtcagttatgcygcccactggcttaggaa’	This study
menA-rev	‘acacactgttctggagcgtttaatggaa’	This study
menC-pKd4-fw	‘tctccccgcacttgccgccagtgacgccacggccagtcagagaagatcatgtgtagg’	This study
menC-pKd4-rev	‘tcaaccagaaacgtcagcctgacttcagcaaattcaaacggaatccgtaaatgggaa’	This study

menC-test-fw	‘agcatttgctccaatggctgt’	This study
menC-test-rev	‘cttgtgaacaccgtggtacc’	This study
menC-fw	‘attctaactacggttactctatgcgtagcgcgcaggtata’	This study
menC-rev	‘tattttacattggcgatgcgtcataacaaccgctccagtgc’	This study
pAF-menC-fw	‘ccaccagtgccggaatagtt’	This study
pAF-menC-rev	‘atcgtccggatcgccattac’	This study
