# Supplementary material for: Electronic control of redox reactions inside Escherichia coli using a genetic module
Source: PLoS One. 2021 Nov 18;16(11):e0258380. doi: 10.1371/journal.pone.0258380 (PMC8601525; doi:10.1371/journal.pone.0258380)
Supplement: S4 Table — (PDF) [file pone.0258380.s005.pdf]

Table S4. gBlocks Used in This Study, Related to the Experimental Procedures

gblock name	Sequence 5’ -> 3’	Reference
p*epcD*-RBS-*cymA*	‘ccgatcttccccatcggtgatgtcggcgatataggcgccagcaaccgcacctgtggcgccggtgatgccggccacgatgcgtccggcgtagaggatcgagatcgatctcgatctaaagccctggatgcaacgacaaaatctgcttatgattttaaagcgtcttacgttcgtgccgtcgcagaccaaacagcaactgctggttatgtaaaaactaaaataatttctattttatattattccctgttttaattaactctatcagggatggtttgtttaactttaagaaggagatatacatattttggagatagagtaatgaactggcgtgcactatttaaacccagcgcgaaatattccatcctagcgctactggttgttggtatcgtgattggtgttgtgggctattttgcaactcagcagactttacatgcgacaagtacagatgcgttctgtatgtcttgccatagcaatcattccttgaagaatgaagtgctggcatctgcccacggtggcggcaaagccggggttactgttcagtgtcaagactgtcacttaccccatggccctgttgattatttaattaagaaaatcatcgtatctaaagatttatatggtttcttaactattgatggctttaacactcaagcttggttagacgaaaaccgcaaagagcaagccgacaaagcattggcttacttccgtggtaacgactcagcaaactgtcaacactgccatactcgcatttatgaaaaccagccagaaaccatgaagccaatggctgtgagaatgcacaccaacaacttcaagaaagatcctgaaacgagaaagacctgtgtggattgccacaaaggtgtcgctcacccctatccaaaaggataaggtttaacgctgcaag’	This study
frdA-crisper	'catggatcctatttcttaataactaaaaatatggtataatactcttaataaatgcagtaatacaggggcttttcaagactgaagtctagctgagacaaatagtgcgattacgaaatttttttagacaaaaatagtctacgaggttttagagctatgctgttttgaatggtcccaaaacatgacccaactggaactgtgggggtgcccagttttagagctatgctgttttgaatggtcccaaaacttcagcacatgaattcaactcaacaagtctcagtg'	This study
