# Supplementary material for: Electronic control of redox reactions inside Escherichia coli using a genetic module
Source: PLoS One. 2021 Nov 18;16(11):e0258380. doi: 10.1371/journal.pone.0258380 (PMC8601525; doi:10.1371/journal.pone.0258380)
Supplement: S1 Raw images — (PDF) [file pone.0258380.s006.pdf]

S1 raw images

Figure S2B

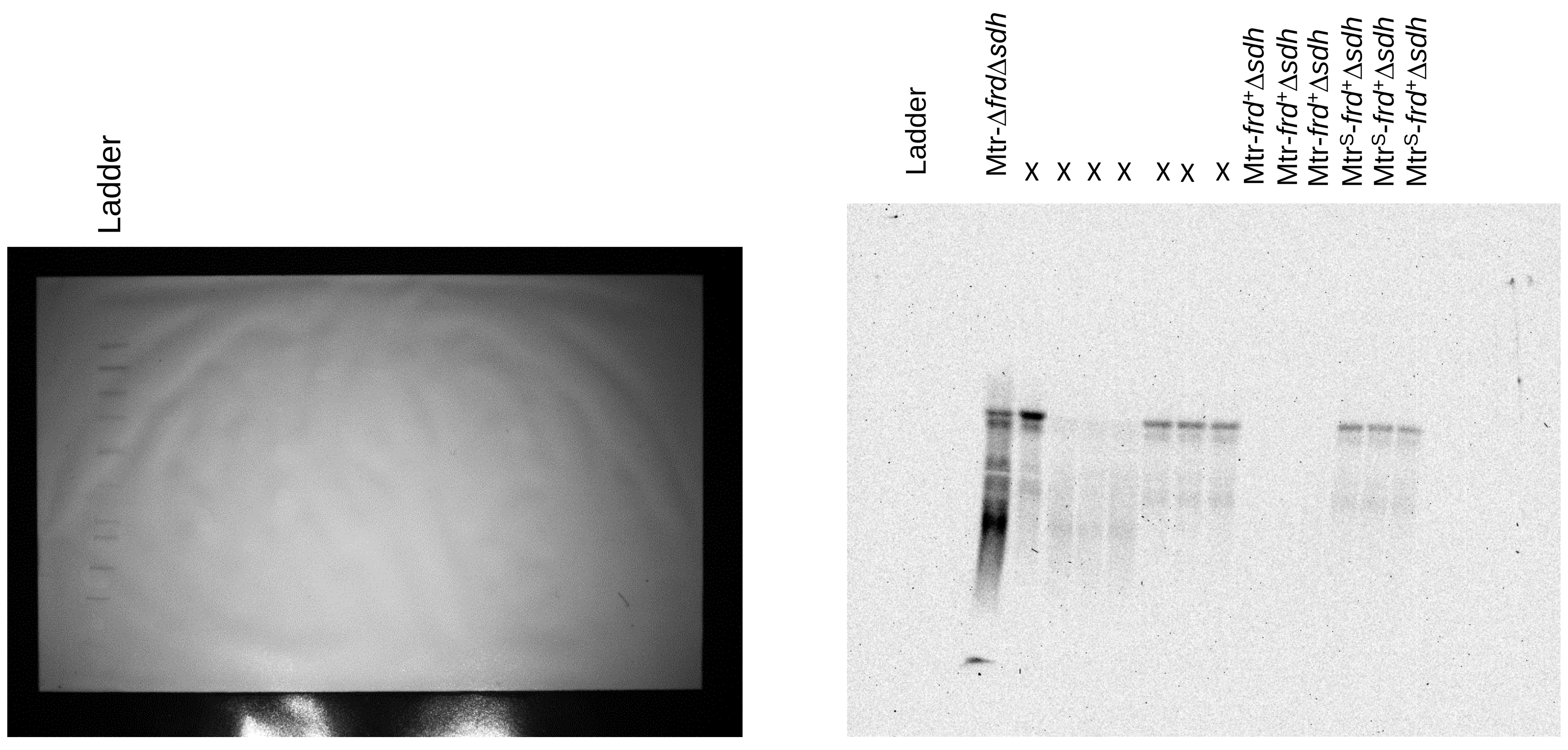

left panel – the ladder (Precision Plus Protein western standard, Biorad) bends can be detected in ambient light. The ladder on the nitrocellulose membrane was capture under white light using CCD imager.

Right panel – the same nitrocellulose membrane, as the one presented in the left panel, was imaged using the Enhanced chemiluminescence (ECL) method.

As the ladder can not be visualized using ECL method, the ladder on the membrane was imaged separately and it image was aligned with the image captured using ECL.

Figure S2C

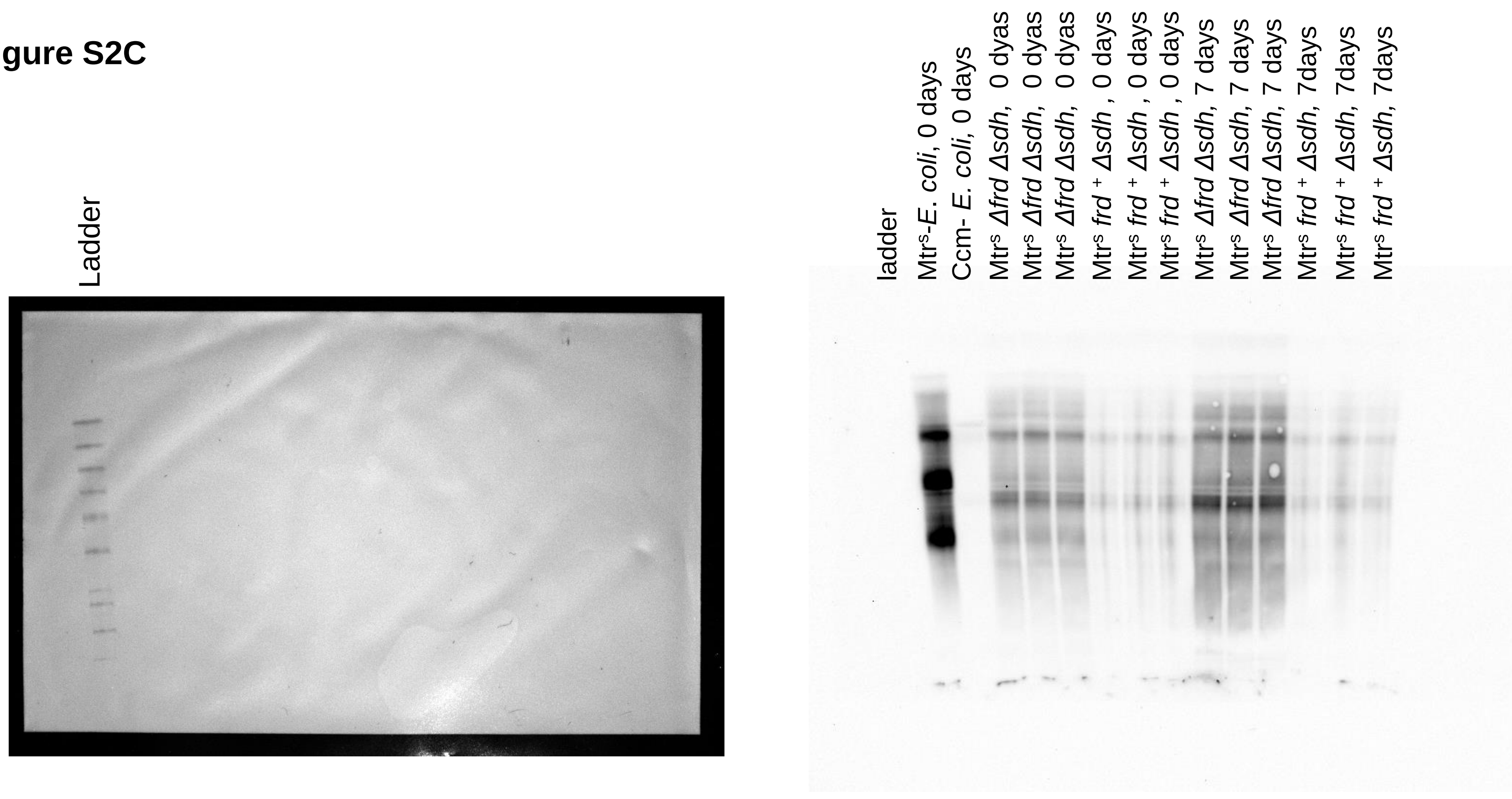

left panel – the ladder (Precision Plus Protein western standard, Biorad) bends can be detected in ambient light. The ladder on the nitrocellulose membrane was capture under white light using CCD imager.

Right panel – the same nitrocellulose membrane, as the one presented in the left panel, was imaged using the Enhanced chemiluminescence (ECL) method.

As the ladder can not be visualized using ECL method, the ladder on the membrane was imaged separately and it image was aligned with the image captured using ECL.

Figure S3B

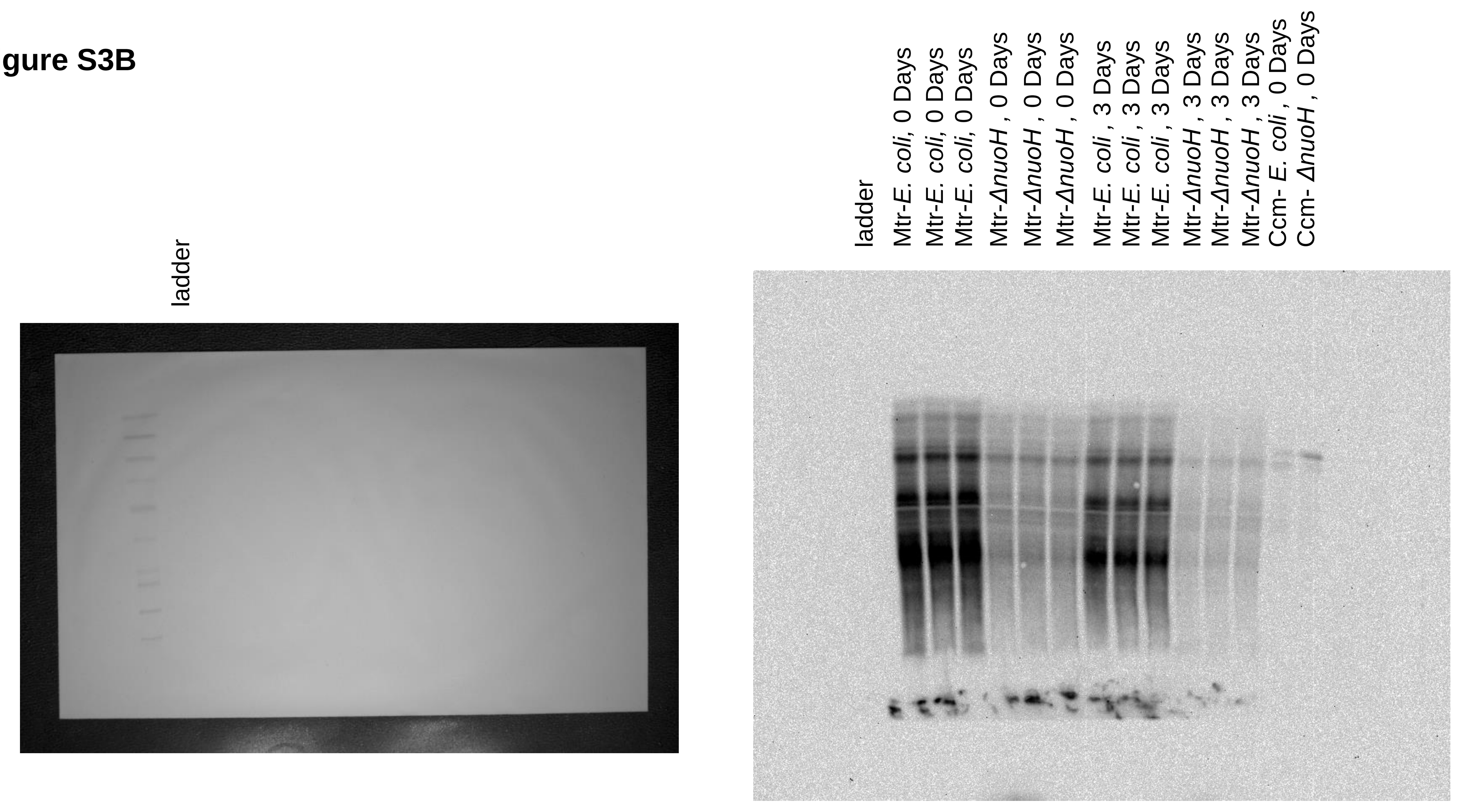

left panel – the ladder (Precision Plus Protein western standard, Biorad) bends can be detected in ambient light. The ladder on the nitrocellulose membrane was capture under white light using CCD imager.

Right panel – the same nitrocellulose membrane, as the one presented in the left panel, was imaged using the Enhanced chemiluminescence (ECL) method.

As the ladder can not be visualized using ECL method, the ladder on the membrane was imaged separately and it image was aligned with the image captured using ECL.

Figure S4C

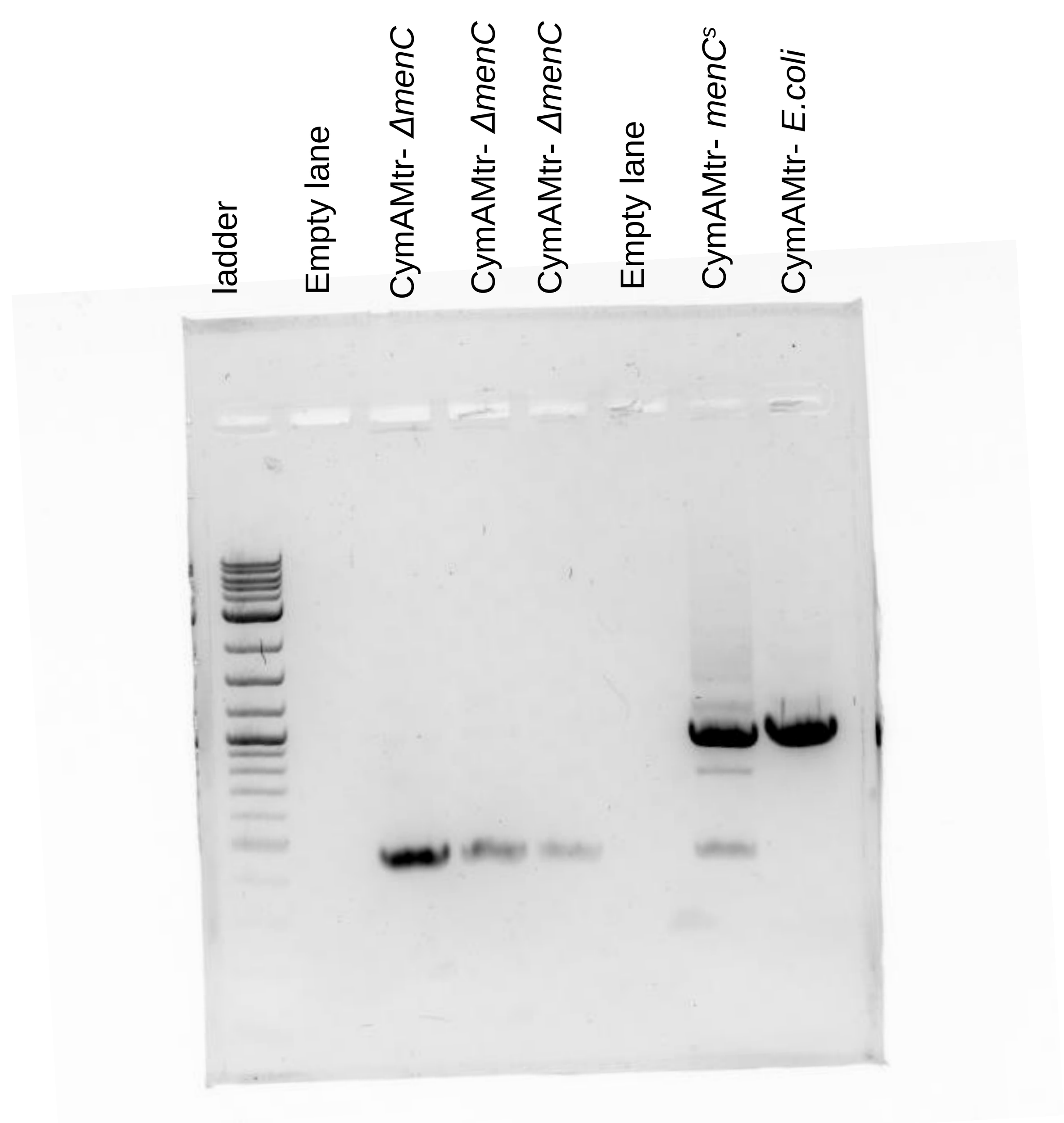

Agarose gel electrophoresis of the PCR products. The gel was stained using SYBR Safe staining and imaged under uv light.
