# Supplementary material for: Electronic control of redox reactions inside Escherichia coli using a genetic module
Source: PLoS One. 2021 Nov 18;16(11):e0258380. doi: 10.1371/journal.pone.0258380 (PMC8601525; doi:10.1371/journal.pone.0258380)
Supplement: S1 Fig — (A) Chronoamperometry upon fumarate addition to abiotic of bioelectrochemical reactors (black) and reactors containing CymAMtr-E. coli (orange), showing no sustained change in current in abiotic reactors. (B-D) Cyclic voltammetry (CV) measurement of bioelectrochemical reactors (B) without E. coli, (C) with the Ccm-E. coli strain, and (D) with the CymAMtr-E.coli. Black and orange lines represent the CV before and after 50 mM fumarate addition, respectively. Only reactors containing the CymAMtr-E. coli show a significant catalytic wave, which is located at -350 mVAg/AgCl. (PDF) [file pone.0258380.s007.pdf]

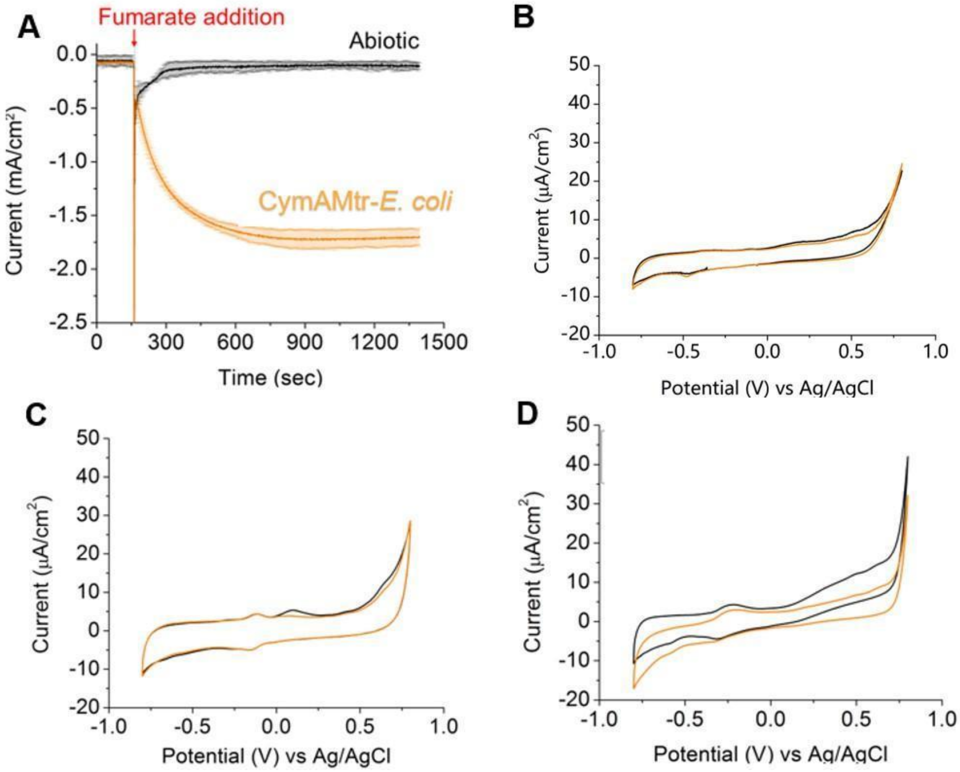

S1 Figure. Electrochemical signatures of biotic fumarate reduction. (A) Chronoamperometry upon fumarate addition to abiotic of bioelectrochemical reactors (black) and reactors containing CymAMtr-*E. coli* (orange), showing no sustained change in current in abiotic reactors. (B-D) Cyclic voltammetry (CV) measurement of bioelectrochemical reactors (B) without *E. coli*, (C) with the Ccm-*E. coli* strain, and (D) with the CymAMtr-*E.coli*. Black and orange lines represent the CV before and after 50 mM fumarate addition, respectively. Only reactors containing the CymAMtr-*E. coli* show a significant catalytic wave, which is located at -350 $mV_{Ag/AgCl}$.
