# Supplementary material for: Electronic control of redox reactions inside Escherichia coli using a genetic module
Source: PLoS One. 2021 Nov 18;16(11):e0258380. doi: 10.1371/journal.pone.0258380 (PMC8601525; doi:10.1371/journal.pone.0258380)
Supplement: S2 Fig — (A) Images of E. coli cell pellets from the CymAMtr-E.coli, CymAMtr-Δfrd Δsdh and CymAMtr frd+Δsdh after aerobic growth in 2xYT in the presence of IPTG. Expression of FrdABCD in the CymAMtr-Δfrd Δsdh mutant results in diminished red color of the bacteria, indicating a low abundance of matured cyts c. (B) Enhanced chemiluminescence (ECL) analysis of cyts c in the CymAMtr-ΔfrdΔsdh, CymAMtr-frd+Δsdh, CymAMtrs-frd+Δsdh after aerobic growth in 2xYT in the presence of IPTG. These data indicate that introduction of a third plasmid to complement frd abrogates expression of the Mtr cyt c. However, regulating transcription of cymAmtrCAB by the dynamic promoter ecpD (Boyarskiy et al., 2016) restores Mtr cyt c expression. (C) ECL analysis of cyts c in the Mtrs-frd+Δsdh and Mtrs-ΔfrdΔsdh just before inoculation into the bioelectrochemical reactors and 7 days after fumarate was added to the reactors. As a control the cyts c expression was examined Mtrs-E. coli and Ccm-E.coli (the two left lanes) which were grown in the same condition as the tested strain pre inoculation. (D) Growth curves of strains grown anaerobically in minimal medium supplemented with non fermentable glycerol, as the electron donor, and fumarate as the electron acceptor. (PDF) [file pone.0258380.s008.pdf]

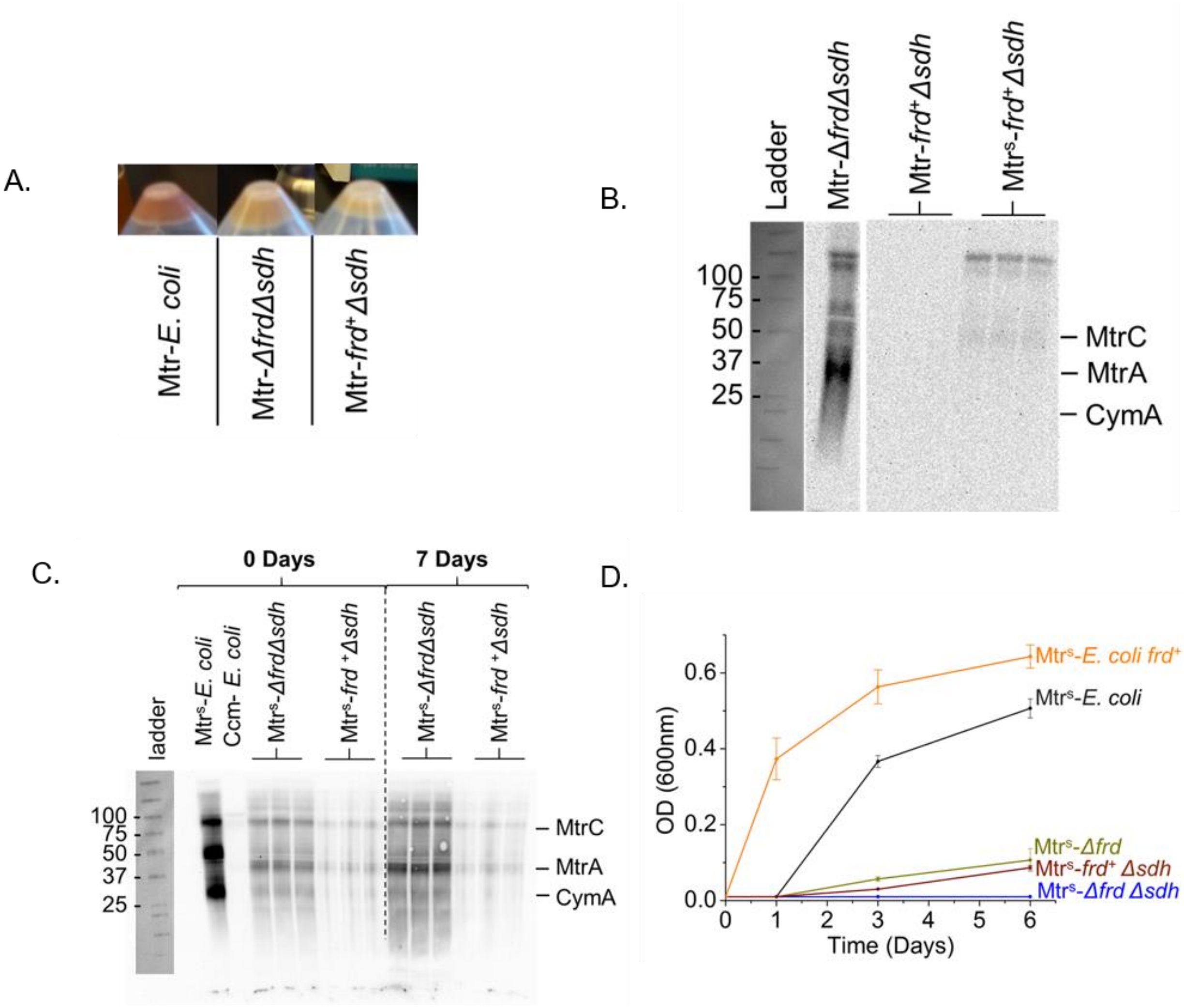

S2 Figure. Heterologous co-expression of CymAMtr and FrdABCD in *ΔfrdΔsdh* mutant. (A) Images of *E. coli* cell pellets from the CymAMtr-*E.coli*, CymAMtr-*Δfrd Δsdh* and CymAMtr *frd*$^{+}$*Δsdh* after aerobic growth in 2xYT in the presence of IPTG. Expression of FrdABCD in the CymAMtr-*Δfrd Δsdh* mutant results in diminished red color of the bacteria, indicating a low abundance of matured cyts *c*. (B) Enhanced chemiluminescence (ECL) analysis of cyts *c* in the CymAMtr-*ΔfrdΔsdh*, CymAMtr-*frd*$^{+}$*Δsdh*, CymAMtr^{s}-*frd*$^{+}$*Δsdh* after aerobic growth in 2xYT in the presence of IPTG. These data indicate that introduction of a third plasmid to complement *frd* abrogates expression of the Mtr cyt *c*. However, regulating transcription of *cymAmtrCAB* by the dynamic promoter ecpD (Boyarskiy et al., 2016) restores Mtr cyt *c* expression. (C) ECL analysis of cyts *c* in the Mtr^{s}-*frd*$^{+}$*Δsdh* and Mtr^{s}-*ΔfrdΔsdh* just before inoculation into the bioelectrochemical reactors and 7 days after fumarate was added to the reactors. As a control the cyts *c* expression was examined Mtr^{s}-*E. coli* and Ccm-*E.coli* (the two left lanes) which were grown in the same condition as the tested strain pre inoculation. (D) Growth curves of strains grown anaerobically in minimal medium supplemented with non fermentable glycerol, as the electron donor, and fumarate as the electron acceptor.
