# Supplementary material for: Electronic control of redox reactions inside Escherichia coli using a genetic module
Source: PLoS One. 2021 Nov 18;16(11):e0258380. doi: 10.1371/journal.pone.0258380 (PMC8601525; doi:10.1371/journal.pone.0258380)
Supplement: S3 Fig — (A) Chronoamperometry of CymAMtrCAB-E. coli upon addition of fumarate after 1 day (black), 3 days (orange), and 7 days (blue) of carbon-source deprivation, showing that increasing starvation also increases current consumption. (B) ECL analysis of the CymA, MtrC, and MtrA abundance in the CymAMtr-E. coli and CymAMtr-ΔnuoH strains when inoculated into the bioelectrochemical reactor (0 days) and 3 days after after addition of fumarate (3 days). As a negative control, the expression level of Ccm-E. Coli and Ccm-ΔnuoH strain is shown. Those strains were grown in the same condition as the tested strain pre-inoculation. (PDF) [file pone.0258380.s009.pdf]

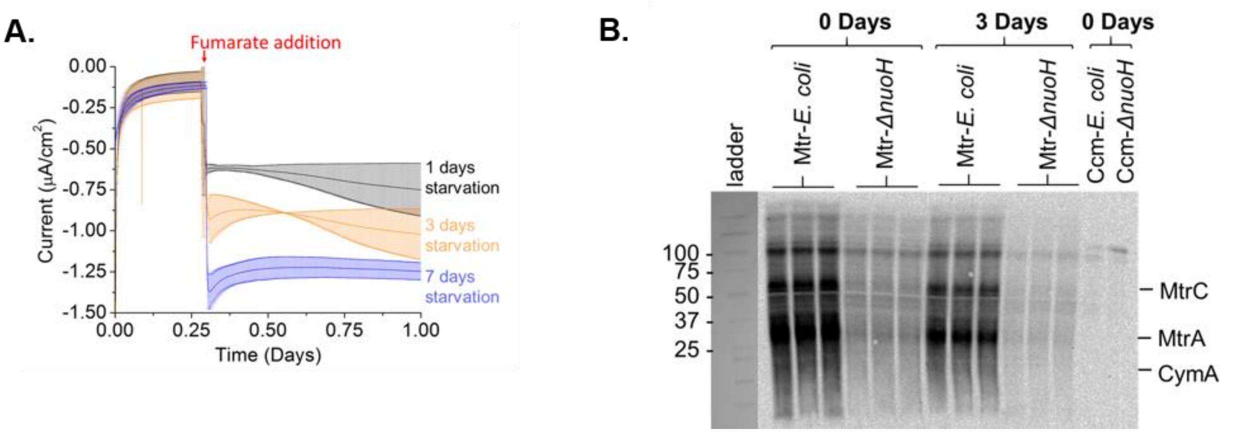

S3 Figure. The influence of starvation on current production and the effect of Complex I disruption on cyt *c* expression. (A) Chronoamperometry of CymAMtrCAB-*E. coli* upon addition of fumarate after 1 day (black), 3 days (orange), and 7 days (blue) of carbon-source deprivation, showing that increasing starvation also increases current consumption. (B) ECL analysis of the CymA, MtrC, and MtrA abundance in the CymAMtr-*E. coli* and CymAMtr-*ΔnuoH* strains when inoculated into the bioelectrochemical reactor (0 days) and 3 days after after addition of fumarate (3 days). As a negative control, the expression level of Ccm-*E. Coli* and Ccm-*ΔnuoH* strain is shown. Those strains were grown in the same condition as the tested strain pre-inoculation.
