# Supplementary material for: Electronic control of redox reactions inside Escherichia coli using a genetic module
Source: PLoS One. 2021 Nov 18;16(11):e0258380. doi: 10.1371/journal.pone.0258380 (PMC8601525; doi:10.1371/journal.pone.0258380)
Supplement: S4 Fig — (A-B) Growth curves of strains grown in an anaerobic in minimal medium containing glycerol as the electron donor. No electron acceptor is provided (A) or Fumarate is provided as an electron acceptor (B). (C) Agarose gel electrophoresis of the PCR products obtained from the genomic DNA from CymAMtr-E. coli, CymAMtr-ΔmenC and CymAMtr-menCS strains. (PDF) [file pone.0258380.s010.pdf]

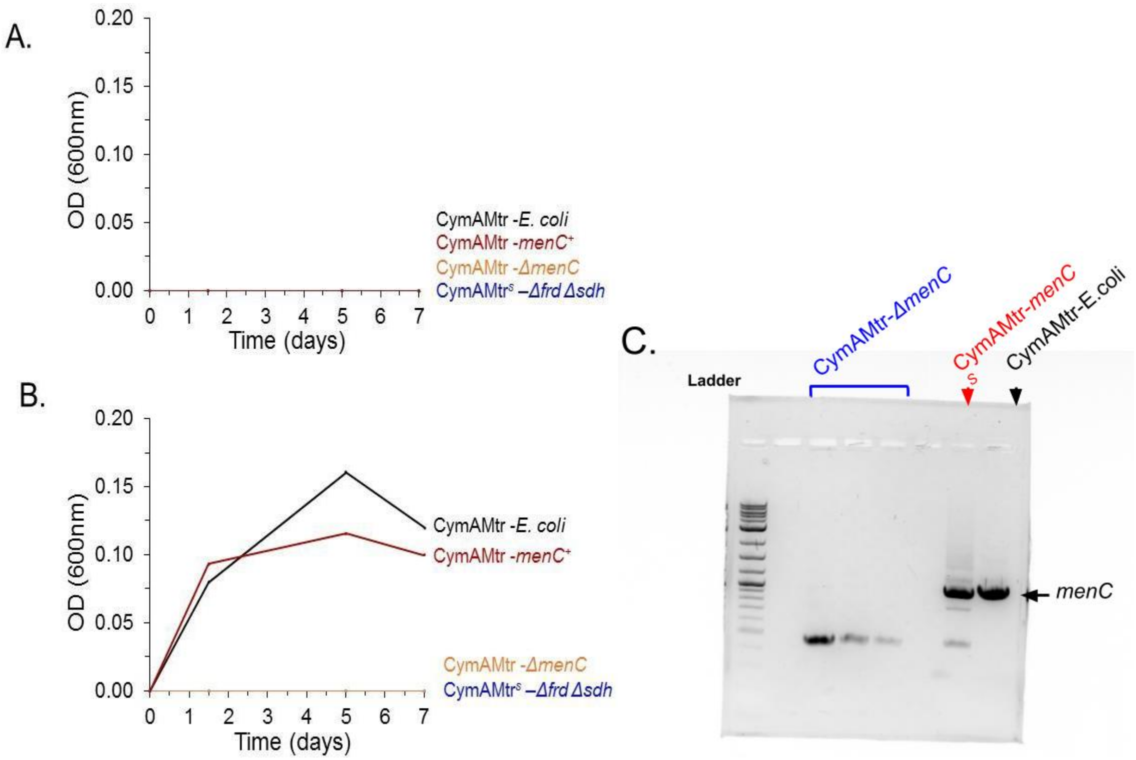

S4 Figure. MenC is essential for fumarate respiration under anaerobic condition in CymAMtr-*E. coli* strain . (A-B**)** Growth curves of strains grown in an anaerobic in minimal medium containing glycerol as the electron donor. No electron acceptor is provided (A) or Fumarate is provided as an electron acceptor (B). (C) Agarose gel electrophoresis of the PCR products obtained from the DNA from CymAMtr-*E. coli*, CymAMtr-*ΔmenC* and CymAMtr-*menC*^{S} strains.
