# Supplementary material for: Electronic control of redox reactions inside Escherichia coli using a genetic module
Source: PLoS One. 2021 Nov 18;16(11):e0258380. doi: 10.1371/journal.pone.0258380 (PMC8601525; doi:10.1371/journal.pone.0258380)
Supplement: S5 Fig — (A) Cyclic voltammetry measurement showing no sustained change in current between abiotic reactors that don’t contain or contain 40mM pyruvate (B) Representative HPLC chromatogram of a sample from the supernatant of a bioreactor after 14 days of CymAMtr-E. coli incubation. The chromatogram displays the peaks of the following analytes: pyruvate, malate, succinate, formate, acetate and fumarate. (C) A plot representing the supernatant acetate, pyruvate malate and fumarate concentration in Bias (Black) vs unbiased (orange) bioreactors containing the Mtr-ΔnuoH mutant. Measurement started upon 50mM Fumarate addition and were taken over a period of 14 days values of 0.0 on plots indicating that the concentration was below the detection limit in that sample. All values are means of triplicate bioreactors, and error bars represent standard error. (D) A plot representing the supernatant succinate, formate, acetate, pyruvate malate and fumarate concentration in Bias(Black) vs unbiased (orange) bioreactors containing the Mtr-E. coli strain. Measurement started upon 50mM Fumarate addition and were taken over a period of 14 days values of 0.0 on plots indicating that the concentration was below the detection limit in that sample. All values are means of triplicate bioreactors, and error bars represent standard error. (PDF) [file pone.0258380.s011.pdf]

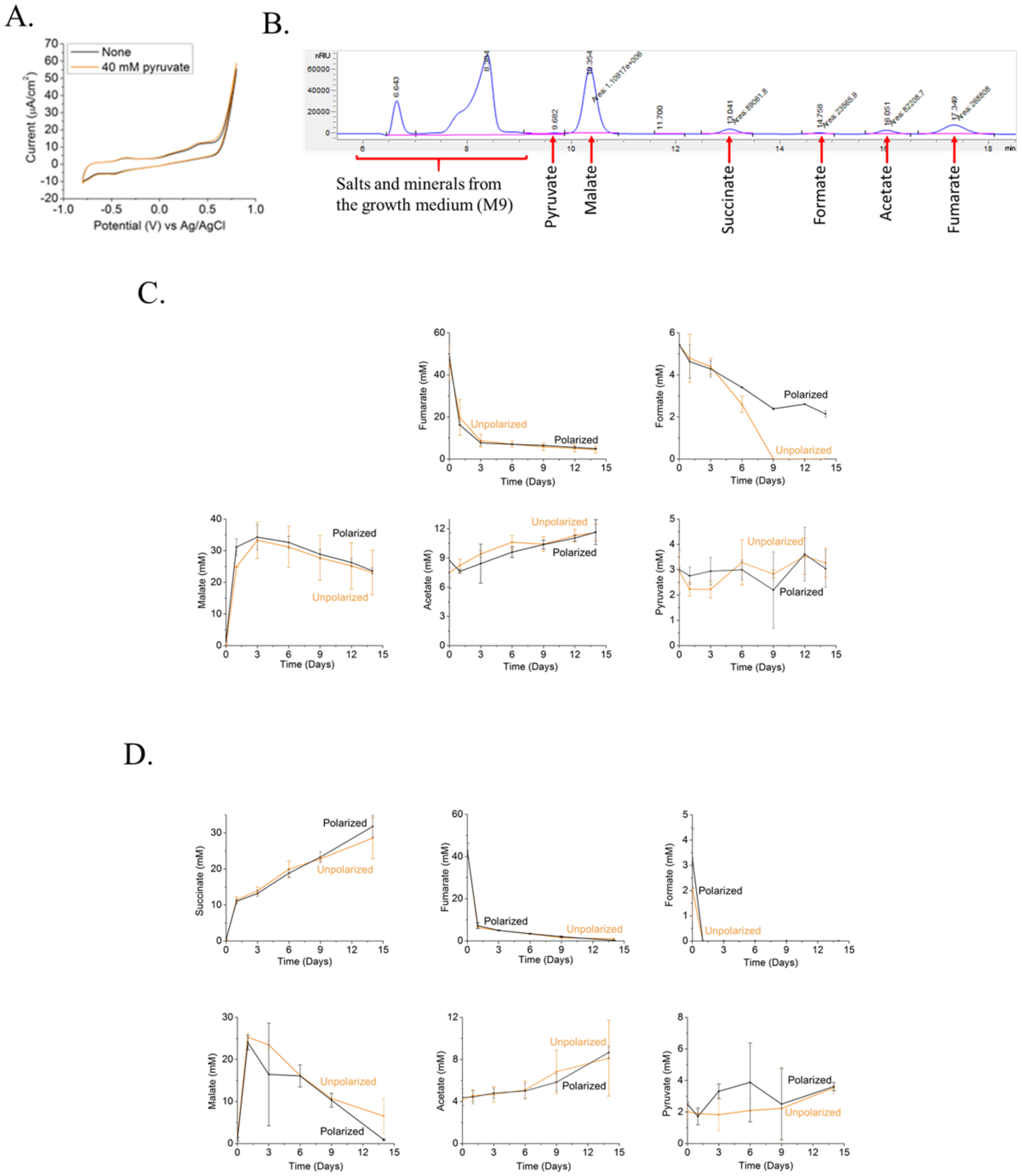

S5 Figure. (A) Cyclic voltammetry measurement showing no sustained change in current between abiotic reactors in the absence or presence of 40 mM pyruvate (B) Representative HPLC chromatogram of a sample from a the supernatant of a bioreactor after 14 days of CymAMtr-*E. coli* incubation. The chromatogram displays the peaks of the following analytes: pyruvate, malate, succinate, formate, acetate and fumarate. (C) A plot representing the supernatant acetate, pyruvate, malate and fumarate concentration in Bias (Black) vs unbiased (orange) bioreactors containing the CymAMtr-*ΔnuoH* mutant. Measurements started upon 50mM fumarate addition and were taken over a period of 14 days values of 0.0 on plots indicating that the concentration was below the detection limit in that sample. All values are means of triplicate bioreactors, and error bars represent

standard error. (D) A plot representing the supernatant succinate, formate, acetate, pyruvate malate and fumarate concentration in Bias(Black) vs unbiased (orange) bioreactors containing the Mtr-*E. coli* strain. Measurements started upon 50mM fumarate addition and were taken over a period of 14 days values of 0.0 on plots indicating that the concentration was below the detection limit in that sample. All values are means of triplicate bioreactors, and error bars represent standard error. Pyruvate (40 mM) was added to the bioreactors to sustain bacterial viability.
