# Supplementary material for: Electronic control of redox reactions inside Escherichia coli using a genetic module
Source: PLoS One. 2021 Nov 18;16(11):e0258380. doi: 10.1371/journal.pone.0258380 (PMC8601525; doi:10.1371/journal.pone.0258380)
Supplement: S6 Fig — Red arrow indicates addition of nitrate to 10 mM, and the error bars indicate the standard deviation in current from triplicate bioelectrochemical reactors. (PDF) [file pone.0258380.s012.pdf]

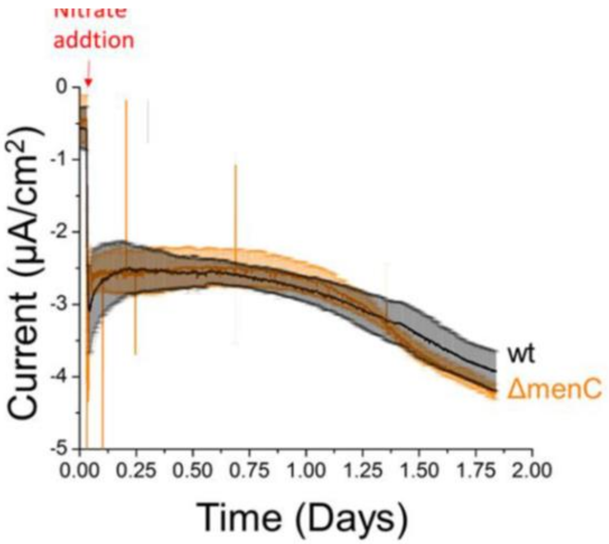

S6 Figure. Chronoamperometry of bioelectrochemical reactors containing CymA-Mtr *E. coli* (black) and CymAMtr-menC (orange) upon nitrate addition, showing no significant change in the current consumed. Red arrow indicates addition of nitrate to 10 mM, and the error bars indicate the standard deviation in current from triplicate bioelectrochemical reactors.
