# Supplementary material for: Electronic control of redox reactions inside Escherichia coli using a genetic module
Source: PLoS One. 2021 Nov 18;16(11):e0258380. doi: 10.1371/journal.pone.0258380 (PMC8601525; doi:10.1371/journal.pone.0258380)
Supplement: Attachment — Submitted filename: Baruch-PlosOneRevision_ResponseReviewers_final.docx [file pone.0258380.s013.docx]

# Response to Reviewer Comments for “Electronic control of redox reactions inside *Escherichia coli* using a genetic module”

## Reviewer #1:

In this work, the authors clearly demonstrate that E. coli modified to express electron uptake pathways from S. oneidensis are functional for reduction of specific electron acceptors. In addition, the work shows that unknown electron carriers and unknown electron sinks within the cell change the stoichiometry of redox reactions and likely affect the redox state of the cell. These are important steps towards understanding how to engineer extracellular electron uptake for biotechnology applications. I found the paper to be well-written and straightforward with the exception of the section describing results with the CymAMtr-ΔnuoH strain. Below are some questions for clarification on this section as well as some minor points.

The results with the CymAMtr-ΔnuoH strain are striking, but also a bit confusing to comprehend. I have a number of questions below for which I would be grateful if the authors would clarify in the text.

We thank the Reviewer for her strong support of our work and her feedback on areas that need additional clarification.

1. Line 270 - The number of c cyts was much lower in the CymAMtr-ΔnuoH strain. Is there a growth defect for this strain? Some reason why disrupting Complex I would affect all c cyt?

The Reviewer is correct that the decrease in cyt *c* expression in the Complex I strain was unexpected. However, it is known that disrupting Complex I can impair growth, particularly as cells approach stationary phase (Prüss, et al. *Journal of Bacteriology*, 1994). Thus, we hypothesize the impaired growth in the Complex I deletion strain affects cyt *c* expression. We have edited the text to clarify this point (lines 451-453).

On line 284 the authors note that they must provide pyruvate to sustain viability in the bioelectrochemical experiments but was pyruvate always added to this strain?

In most experiments with the CymAMtr-Δ*nuoH* strain, we did not add pyruvate. Rather, we added pyruvate only when we studied succinate and ammonia accumulation (Figure 5) because these experiments required 14 days.

We have edited the text to clarify this point (lines 263-264).

I may just be naïve about E.coli growth metabolism but if you take out Complex I don’t you have to grow fermentatively?

Yes, in the absence of Complex I, *E. coli* will mainly grow by fermentation.

2. Line 273-274 – This statement is a bit confusing. Are the authors trying to say that electrons from reduced NADH normally enter Complex I and provide reducing equivalents for fumarate reduction during electron uptake from the cathode? Where are the electrons coming from? Pyruvate fermentation?

We thank the Reviewer for pointing out the lack of clarity here, which Reviewer #2 also indicated.

CymAMtrA-*E. coli* produce current for several days in the absence of an electron donor (ref 16 and Fig. S3), indicating this strain stores reducing equivalents under our growth conditions. We suggest that, under cathodic conditions, these stored reducing equivalents can be passed via Complex I to menaquinone and subsequently fumarate reductase, thus competing with electrons from the cathode. We have clarified this in the manuscript (lines 421-430).

The experiments in Figure 4 do not contain pyruvate, so pyruvate fermentation cannot be a source of reducing equivalents.

3. Line 282 – Was the CymAMtr-ΔnuoH strain used for subsequent fumarate/succinate stoichiometry because it had higher electron uptake?

Yes, we chose to use this strain because of its higher current consumption capacity. We have clarified this reasoning in the manuscript (lines 463-464).

4. Line 290 – I think I am missing something…what does it mean that succinate consumption is “equal under polarized and unpolarized conditions”? Succinate is coming from fumarate so is it also the assumption that fumarate is reduced equally? I think this gets back to my question 3 above. Is there an electron donor in the system other than the electrode? Are the authors assuming pyruvate?

We thank the Reviewer for this comment and have edited this paragraph to clarify our reasoning (lines 476-479).

We assumed that succinate consumption was equal under both conditions so that we could interpret measured differences in succinate concentration as arising from differences in succinate production. This assumption made it possible to calculate how the amount of current consumed would change the succinate concentrate.

In fact, the calculations of how the succinate concentration would change are not consistent with the observed data. Therefore, we speculate that one possibility for the non-stoichiometric accumulation of succinate is differences in its consumption between the polarized and unpolarized conditions (lines 499-501).

Minor points
Line 58 – do authors mean -560 mV?

Yes. We corrected this error (line 60).

Line 114 – Can authors clarify the difference between a technical and biological replicate? The figures show “replicates” but it is not clear what this means based on this statement in the methods.

Biological replicates are experiments employing a different culture of the same strain. Technical replicates are experiments using the same culture in different bioelectrochemical reactors. We have clarified this distinction in the methods (lines 264-266).

Line 132 – missing word?

We have corrected this typo.

Line 139 – references Fig 1A but that is not what is depicted.

We apologize for this mistake. We have corrected this statement to refer to Figure 1B (line 313-14).

Line 146 – It isn’t really clear why strains need to be grown anodically before testing cathodically. Is this just to validate EET?

The Reviewer raises a good point, and we have now explained this point in the manuscript (lines 322-325). We exposed cells to an anode first for two reasons: a) to exhaust any possible reducing equivalents stored in the cells to minimize competing electron donors to the cathode and b) to promote cell attachment to the electrode. We also found this acclimating period was critical to obtain reproducible results.

Line 157 – Are you sure there are no changes in gene expression? What about the fumarate reductase?

We thank the Reviewer for this important comment. We do not have any direct evidence that there are no changes in gene expression. Rather, we have indirect evidence that gene expression is not required for current consumption: only ~30s elapsed between fumarate addition and measurable current consumption, which is far too rapid for meaningful gene expression. However, it is entirely possible that gene expression occurs on a longer timescale and contributes to sustaining current. We have edited the manuscript to reflect this point (lines 334-335).

Line 163 – C43(DE3) is not really described before this point and the reader is left to assume that this is the strain used here.

We thank the Reviewer for this point. We have included this information in the last paragraph of the Introduction (lines 83).

## Reviewer #2:

Baruch et al. investigate electron uptake in E. coli engineered to express Mtr pathway components from S. oneidensis. Electron uptake to fumarate and nitrate could be demonstrated, the latter case seems to be stoichiometric conversion to ammonia (which is nice!). One of the major challenges in this system is the very low levels of current produced / consumed and the very large number of planktonic cells (0.6 OD). Overall, the findings are interesting and the work is sound. A better job can be done with the background on the known EET pathway of S. oneidensis. I would also like to see a little more detail in the main text regarding the claim of stoichiometric conversion of nitrate to ammonia. Comments for the authors consideration are below.

We thank the Reviewer for the Reviewer’s endorsement of our work and helpful feedback on areas for improvement.

Line 1 – the authors may wish to provide a more accurate title. I don’t see how this control is ‘precise’ in nature.

We agree with this feedback and have eliminated the word ‘precise’ from the title (line 1).

Line 27 –Fumarate is intracellular, but nitrate is periplasmic.

In this particular case, nitrate is also intracellular. In the *E. coli* C43(DE3) background, the genes for the periplasmic nitrate reductase NapFDAGHBC are deleted, while the membrane-bound respiratory reductase NarGHI remains intact (ref. 37). Since the active site of the NarGHI reductase is located in the cytoplasm (ref. 48), nitrate reduction in this background is expected to occur exclusively intracellularly. We have included this important nuance in the manuscript (lines 509-517).

Line 53 – the conversion of lactate to pyruvate will send electrons directly into the quinone pool or will produce NADH (depending if D-lactate or L-lactate is being metabolized). Conversion of pyruvate to acetate generates formate anaerobically, not NADH.

We apologize for this misstatement and have corrected it in the manuscript (lines 54-56).

Line 56 – both CctA (also known as Stc) and FccA move electrons between CymA and MtrCAB. See reference 22 here. Mutants lacking cctA have no discernable metal or electrode reduction phenotype.

We apologize for this oversimplification and have clarified that both Stc and FccA are periplasmic electron carriers (line 57).

Line 59 – the references here are confusing. One reference shows electron uptake to oxygen and the other to fumarate under anoxic conditions?

The Reviewer is right. We have corrected the reference (line 61).

Figure 1B – FccA does not directly interact with the menaquinone pool. It received electrons from CymA.

We have corrected the schematic so that it is clear that FccA receives electrons from CymA (revised Figure 1).

Line 76 – Unclear why differences in OM permeability (the references suggests, but does not demonstrate, that B strains have larger OM porins) would influence electron transfer across the outer membrane. The authors have not described why type II secretion is important to electron transfer.

The Reviewer raises a good point. Previous work in *E. coli* has correlated increased OM permeability to increased extracellular electron transfer via electron shuttles (C. Catania, C. M. Ajo-Franklin, G. C. Bazan, *RSC Adv.* **6**, 100300–100306 (2016); Y.-C. Yong, et al., *Biotechnol. Bioeng.* **110**, 408–416 (2013).). Type II secretion is important because it is required to localize outer membrane cytochromes to the outer membrane and for insoluble iron reduction (ref. 30). We have added these points to the manuscript (lines 79--83).

Line 83 – why not include all methods here for completeness? Is this a space constraint from a past submission?

We have followed the Reviewer’s recommendation and included all of the methods relating to the Figures in the main manuscript (lines 88-111, 149-243, 285-308). We retained the methods specific to SI Figures in the SI.

Line 146 – unclear why anaerobic, anodic incubation is required to prepare E. coli for cathodic conditions.

Reviewer 1 also commented on this omission, and we apologize for the lack of clarity here. We have included this clarification in the manuscript (lines 322-325). We exposed cells to an anode first for two reasons: a) to exhaust any possible reducing equivalents stored in the cells to minimize competing electron donors to the cathode and b) to promote cell attachment to the electrode. We also found this acclimating period was critical to obtain reproducible results.

Line 162 – is it appropriate to cite an un-reviewed biorxiv paper?

We understand the Reviewer’s concern. This reference just came out in print and we have updated it accordingly (ref 36).

Line 163 – the reference cited here does not provide information related to NapC complementing CymA.

The reference provided supports the statement that NapC is disrupted in the C43(DE3) background. We have rephrased this in the manuscript to clarify this point (lines 340-341).

Line 186 – was a CymAMtr strain with sdh missing also tested (I don’t’ think it need to be, but I’m curious)? Did these new mutant strains exhibit any phenotype on the anode, assuming they were pre-grown in this fashion as previously done?

We did not prepare a CymAMtr strain with sdh knockout for this paper. We appreciate the Reviewer’s suggestion and may include it in future studies.

Line 207 – unclear why the authors are concerned about polarity given that these are deletion mutants? Could this figure be moved to supplemental to help focus the work?

We were concerned that deletion of *frd* and *sdh* had caused changes in other pathways that could impact EET. Thus, we find this complementation experiment a necessary test to prove that the disruption of current consumption was exclusively due to the removal of only *frd* and *sdh.* We have removed the words ‘polar effect’ to clarify this point (lines 382-383, lines 389-390).

Line 234 – you aren’t comparing current production to wild-type – be specific!

Thank you for this correction. We have replaced ‘wild type’ by CymAMtr*-E.coli* (line 410).

Line 239 – CymA in S. oneidensis requires a MK co-factor to function and is also required to reduce FccA, the periplasmic fumarate reductase. It doesn’t seem surprising to me that the menaquinone mutants still exhibited cathodic fumarate reduction. There is likely reduced ubiquinone that is facilitating reduction of the reductase complexes.

We agree with the Reviewer here and have removed the word ‘surprising’ (line 412).

Line 240 – unless there is free MtrA in the periplasm for some reason, considering the structure of the MtrCAB complex, it should not be able to get anywhere near cytoplasmic membrane complexes like Fdh and Sdh.

Prior work with this *E. coli* strain showed that free MtrA (without MtrC) can be extracted from the periplasm (ref. 17), which makes it more likely that it could interact with Fdh or Sdh. Therefore, we prefer to leave both hypotheses, direct transfer and biomolecule, in the manuscript. We have edited the manuscript to indicate that MtrA is free in the periplasm (lines 414-5).

Line 271 – specify what ‘Mtr cyt c’ is being referred to here.

We have clarified this in the manuscript. Now it refers specifically to MtrA and MtrC (lines 450).

Line 370 – is E. coli a ‘novel microorganism’?

We agree with the Reviewer on this point and have edited the text to highlight that *E. coli* is an industrially relevant host (line 570-1).

Line 378 (and 416) – the authors have presented two cases for driving reductive reactions. One case was driven stoichiometrically and the other was not. For the nitrate to ammonia example, the authors need to better walk through the math to convince the reader that it is indeed stoichiometric. How much ammonia was generated? Seems like ~ 0.35 mM. How many electrons would need to be consumed to produce this? How much current was in fact consumed over this time?

This point is a key finding of the paper, and we thank the Reviewer for requesting more information here.

The calculations depend critically on our assumption of how many cathodic electrons are required for reduction of nitrate to ammonia. Given that reduction of nitrate to nitrite by NarGHI is MK-linked, this reduction is expected to use two cathodic electrons. However, the Nir complex utilizes NADH as its native electron donor. Data in this manuscript strongly suggests cathodic electrons cannot substitute for NADH. (Addition of pyruvate, which can be reduced to lactate by lactate dehydrogenase using NADH, does not induce consumption of current.) Using the assumption that only the reduction of nitrate to nitrite is driven by cathodic electrons, the estimated and observed change in ammonia concentration are in excellent agreement, 0.49 mM compared to 0.40 ± 0.09 mM. If we assume that all eight electrons required to produce nitrate are provided by the cathode (in contrast to our prior observations), the estimated ammonia concentration is approximately 1/3 what is observed. Therefore, our data indicates nitrate is stoichiometrically reduced to nitrite by the cathode.

We have modified the text to indicate nitrate reduction, rather than ammonia production is stoichiometric (lines 31, 503-504, 519-520, 568-9, 579). We have also highlighted the implication that cathodic electrons cannot serve as electron donors in place of NADH in our pyruvate experiments (lines 471-474), moved the detailed description of the calculations to main text (lines 293-308), and summarized these calculations more completely in the Results (lines 554-566).

In the course of these revisions, we also removed references to experiments examining nitrite reduction. We made these edits because the data was not provided in the original manuscript and because regulation of the nitrite reductases will be substantially different in those experimental conditions and the conditions for Figure 5B, making that data irrelevant to understanding Figure 5.

Line 393 – the heading here seems unnecessary

We have followed the Reviewer’s recommendation and have removed this heading.

Line 410 – what is the evidence that supports the statement here, that MtrA is more abundant than MtrC in this system?

The Reviewer raises a good point here. The heme staining measurements of this strain are suggestive, but not conclusive of this point. Thus, we have removed this statement from the manuscript.

Figure 6 – is this figure useful? Also, I’m pretty sure the nitrate / nitrite reactions occur in the periplasm, not the cytoplasm.

We think Figure 6 is useful to summarize the information we obtained in our study about the mechanism behind the transport of electrons from and to the cytoplasm in Mtr*-E.coli* in the presence of an anode and a cathode, respectively. We believe this Figure will help the reader to better understand the mechanisms involved. We now include additional data showing that the electron transfer route is similarly menaquinone-independent when nitrate or fumarate triggers current consumption (new Fig S6) to better support Figure 6.

As mentioned previously, the only active nitrate reductase in *E. coli* C43(DE3) is NarGHI (ref. 37) and its active site for nitrate reduction is in the cytoplasm (ref. 48). Thus, nitrate reduction in the background used for this study is expected to occur intracellularly.

Table S1 – please add the parent strain (and its complete genotype) to this list.

We added this information to Table S1.

References – missing italics throughout, some are incomplete.

We have reviewed the list of references and corrected the style.

# Response to Editorial requests

When submitting your revision, we need you to address these additional requirements.

1. Please ensure that your manuscript meets PLOS ONE's style requirements, including those for file naming.

Our manuscript now meets PLOS ONE’s style requirements.

2. Please review your reference list to ensure that it is complete and correct.

The reference list in our manuscript is complete and correct. We do not cite any papers that have been retracted. Any changes to the reference list have been included in the reference list.

3. Please amend the manuscript submission data (via Edit Submission) to include author Lin Su.

We have corrected this omission.

4. We note that you have included the phrase “data not shown” in your manuscript. Unfortunately, this does not meet our data sharing requirements. PLOS does not permit references to inaccessible data. We require that authors provide all relevant data within the paper, Supporting Information files, or in an acceptable, public repository.

We edited the manuscript to now include this data as Figure S5 and now refer to this data.

5. Please include captions for ALL your Supporting Information files at the end of your manuscript, and update any in-text citations to match accordingly. Please see our Supporting Information guidelines for more information: http://journals.plos.org/plosone/s/supporting-information.

All captions are included as required.

6. PLOS ONE now requires that authors provide the original uncropped and unadjusted images underlying all blot or gel results reported in a submission’s figures or Supporting Information files. In your cover letter, please note whether your blot/gel image data are in Supporting Information or posted at a public data repository, provide the repository URL if relevant, and provide specific details as to which raw blot/gel images, if any, are not available. Email us at [plosone@plos.org](mailto:plosone@plos.org) if you have any questions.

The original uncropped and unadjusted gel images are provided in the SI.
